# [Corrigendum] Differential regulation of mitochondrial complex I and oxidative stress based on metastatic potential of colorectal cancer cells

**DOI:** 10.3892/ol.2023.13888

**Published:** 2023-05-30

**Authors:** Neeraj Kumar Rai, Shashank Mathur, Suraj Kumar Singh, Meenakshi Tiwari, Vijay Kumar Singh, Rizwanul Haque, Swasti Tiwari, Lokendra Kumar Sharma

Oncol Lett 20: 313, 2020; DOI: 10.3892/ol.2020.12176

Subsequently to the publication of the above paper, an interested reader drew to the authors’ attention that there was a similarity between the loading control in Fig. 3E on p. 6 (the VDAC data) and Fig. 6B on p. 8 (the β-actin bands for the ‘SOD1/Beclin-1/ATG5’ experiment). The authors referred back to their original data and realized that an inadvertent error had been made while assembling the VDAC loading control data in Fig. 3E. The corrected version of Fig. 3E, showing the correct VDAC control is presented below.

The authors deeply regret this minor error, and can confirm that this error did not affect the conclusions reported in the paper. The authors are grateful to the Editor of *Oncology Letters* for granting them the opportunity to publish this Corrigendum, and all the authors agree to its publication. Furthermore, the authors also apologize to the readership for any inconvenience caused.

## Figures and Tables

**Figure 3.**
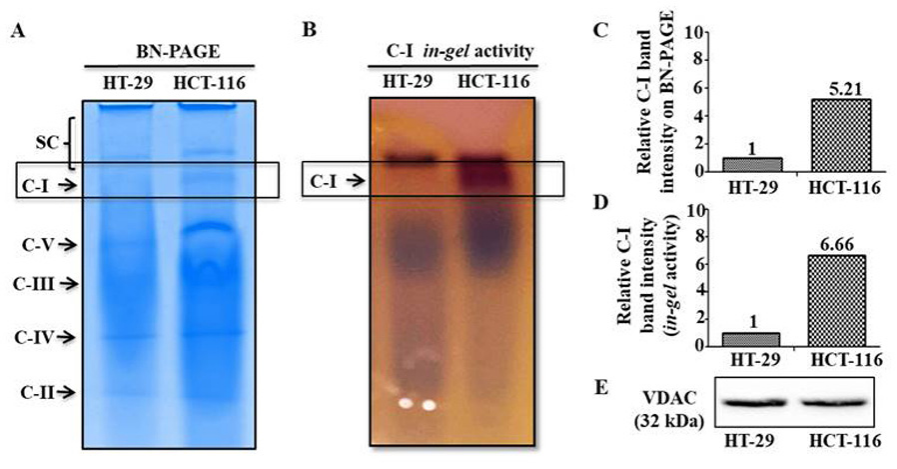
Mitochondrial C-I assembly and activity analysis. (A) BN-PAGE was performed in the isolated mitochondrial preparation of cultured cells. (B) C-I activity was measured using in gel C-I activity assay. Relative band intensities of C-I on (C) BN-PAGE and (D) in-gel assay were quantitated using ImageJ and normalized with total mitochondrial protein loaded (80 µg). (E) Equal loading of mitochondrial proteins is shown by immunoblotting results of an aliquot of the same samples used for BN-PAGE/in-gel assay, with mitochondrial marker protein VDAC. C-, complex; VDAC, Voltage-dependent anion channel; BN-PAGE, Blue native polyacrylamide gel electrophoresis.

